# Improved Database Filtering Technology Enables More Efficient Ab Initio Design of Potent Peptides against Ebola Viruses

**DOI:** 10.3390/ph15050521

**Published:** 2022-04-24

**Authors:** Thomas Ripperda, Yangsheng Yu, Atul Verma, Elizabeth Klug, Michellie Thurman, St Patrick Reid, Guangshun Wang

**Affiliations:** Department of Pathology and Microbiology, College of Medicine, University of Nebraska Medical Center, 985900 Nebraska Medical Center, Omaha, NE 68198-5900, USA; ripperdatj@gmail.com (T.R.); yangshengyu@unmc.edu (Y.Y.); atul.k.verma@hotmail.com (A.V.); liz.klug@unmc.edu (E.K.); michelliethurman19@gmail.com (M.T.)

**Keywords:** antimicrobial peptide database, antiviral peptides, database filtering technology, SARS-CoV-2, Ebola virus, peptide design

## Abstract

The rapid mutations of viruses such as SARS-CoV-2 require vaccine updates and the development of novel antiviral drugs. This article presents an improved database filtering technology for a more effective design of novel antiviral agents. Different from the previous approach, where the most probable parameters were obtained stepwise from the antimicrobial peptide database, we found it possible to accelerate the design process by deriving multiple parameters in a single step during the peptide amino acid analysis. The resulting peptide DFTavP1 displays the ability to inhibit Ebola virus. A deviation from the most probable peptide parameters reduces antiviral activity. The designed peptides appear to block viral entry. In addition, the amino acid signature provides a clue to peptide engineering to gain cell selectivity. Like human cathelicidin LL-37, our engineered peptide DDIP1 inhibits both Ebola and SARS-CoV-2 viruses. These peptides, with broad antiviral activity, may selectively disrupt viral envelopes and offer the lasting efficacy required to treat various RNA viruses, including their emerging mutants.

## 1. Introduction

Emerging viral infections can cause harm to our society. This was made crystal clear in the previous Spanish flu and the current severe acute respiratory syndrome coronavirus 2 (SARS-CoV-2) pandemic. According to the World Health Organization, COVID-19 has caused 464 million infections and 6.06 million cumulative deaths globally as of March 21, 2022 [1]. SARS-CoV-2 has also added a burden to our economic, educational, and health care systems around the world. Similarly, Ebola viruses have a major impact on our society, causing a significant number of mortalities. Ebola viruses are highly lethal and have an average mortality rate of 50% [2,3]. Fortunately, vaccines have proven to be effective to protect humans from SARS-CoV-2 and Ebola virus (EBOV) infections [4,5,6]. However, viruses mutate rapidly and have evolved into numerous mutants that could compromise vaccine protection and cause breakthrough coronavirus infections [7]. Hence, there is a need to develop alternatives such as antiviral drugs to better manage viral infections. 

Antimicrobial peptides (AMPs) are an important factor of the innate immune defense for both invertebrates and vertebrates, including humans [8,9,10,11,12,13,14]. Recently, human defensins have been shown to inhibit SARS-CoV-2 infection [15]. Likewise, human cathelicidin LL-37, another key AMP, also demonstrated antiviral activity against SARS-CoV-2 [16]. These antiviral peptides can work through different mechanisms, ranging from immune regulation to direct inactivation via membrane disruption [17,18,19,20]. Hence, natural AMPs may be engineered into new therapeutics to help control these viruses [11,12,21,22,23]. As a proof of concept, we previously demonstrated that LL-37 could be engineered to inhibit Ebola virus entry [24]. 

As an alternative approach, we found it useful to discover novel antimicrobials based on the antimicrobial peptide database (APD) [25]. This was made possible due to our systematic classification of AMPs based on the sequence length, net charge, hydrophobic ratio, post-translational modification, structure, activity, and source organism. In the APD, most of the AMPs had a peptide length of less than 60 amino acids. The net charge of a peptide was calculated at pH 7 and chemical modification was considered. The majority of the peptides in the APD have a net charge in the range of +1 to +7. The hydrophobic amino acids were defined based on the Kyte–Doolittle hydrophobic scale [26], where residues of leucine (L), isoleucine (I), valine (V), alanine (A), methionine (M), cysteine (C), and phenylalanine (F) are hydrophobic. In addition, the APD included tryptophan (W) in the hydrophobic group. The hydrophobic ratio of a peptide was calculated based on the sum of all the hydrophobic amino acids mentioned above divided by the peptide length. The dominant hydrophobic ratios for AMPs are located between 20 and 70%. Peptide structures are separated into four classes based on the presence or absence of the α-helix and β-sheet (i.e., α, β, αβ, and non-αβ). In the current APD, 478 peptides are found to have an α-helical structure based on nuclear magnetic resonance (NMR) spectroscopy and/or circular dichroism (CD). Only 88 peptides are determined to have a β-sheet structure, while 116 entries are known to adopt an αβ fold. Finally, the structures of 22 peptides are found to belong to the non-αβ class (i.e., no α-helix nor β-sheet). Both the peptide sequence and post-translational modification play an important role in determining 3D structure and biological activity (e.g., antibacterial, antiviral, antiparasitic, antifungal, and anticancer) [27]. 

This study reports the design of novel antiviral peptides by developing an improved version of the database filtering technology [28], making peptide design more efficient. Following up on our initial observation of Ebola virus entry inhibition, we validate the utility of the database filtering approach in designing new antiviral peptides using an Ebola virus pseudo-type system. In addition, we also evaluate the antiviral effects of DDIP1, a database-designed inhibitory peptide 1 [27], using both Ebola virus and SARS-CoV-2. 

## 2. Results and Discussion 

### 2.1. Peptide Design via an Improved Database Filtering Technology

**Figure 1:**
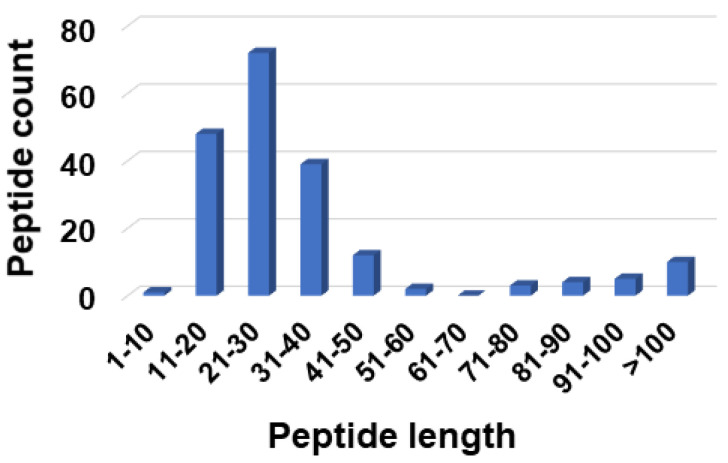
Antiviral peptide count in the antimicrobial peptide database at various peptide length ranges [25]. Peptides in the range of 21–30 amino acids were dominant. This length range would be the most probable length based on our previous design idea [28]. To reduce peptide cost, we selected a peptide length of 20 in this study and two even shorter peptides were also designed based on the peptide parameters with 11–20 amino acids.

**Figure 2:**
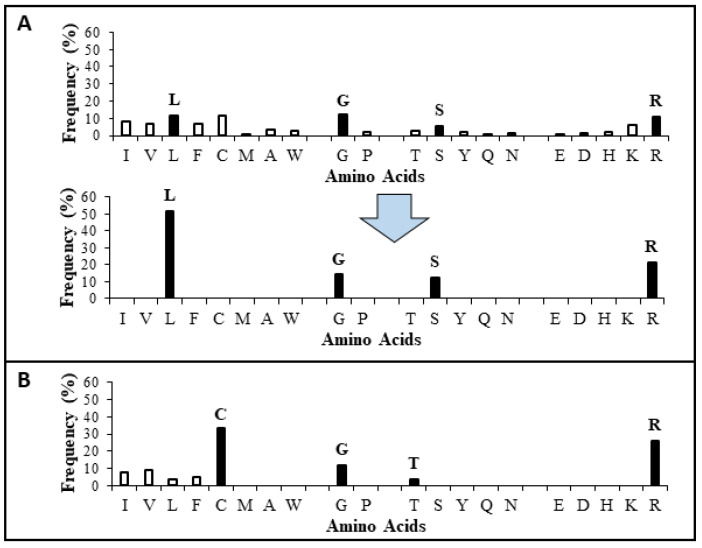
A single-step amino acid analysis of antiviral peptides with 11–20 amino acids to derive multiple most probable parameters for peptide design. (**A**) Four frequent amino acids (L, G, S, and R, solid column) were identified from the four groups of amino acids. The percentages of amino acids for each group were then merged into L, G, S, and R. (**B**) The amino acid analysis of 19 θ-defensins registered in the APD. It is remarkable that in nature, θ-defensins are designed in a similar way by mainly using C, G, T, and R dotted with a few other hydrophobic amino acids.

**Figure 3:**
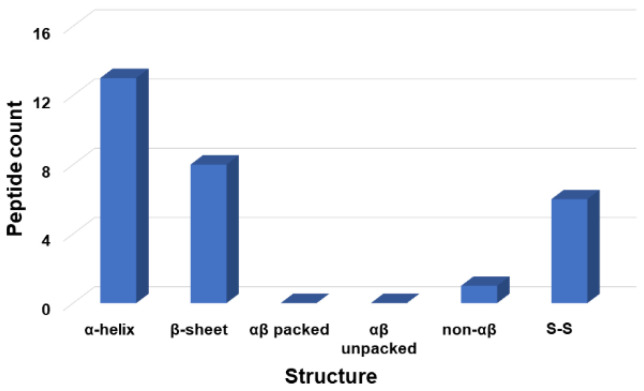
Count of antiviral peptides with different structures. Data were obtained from the antimicrobial peptide database [25] for antiviral peptides with 11–20 amino acids. Antiviral peptides with an α-helical structure were dominant with 13 counts. In addition, there were eight antiviral peptides in the selected length group with β-sheet structures. No antiviral peptides in this length group had a determined αβ structure (packed or unpacked). Only one antiviral peptide in this group had a non-αβ structure. In addition, six antiviral peptides in this group were disulfide-linked (S-S), although their 3D structures are unknown. Note that a similar plot was obtained for antiviral peptides with 21–30 amino acids (not shown). Therefore, α-helical structure was decided as the most probable structure for this design.

**Figure 4:**
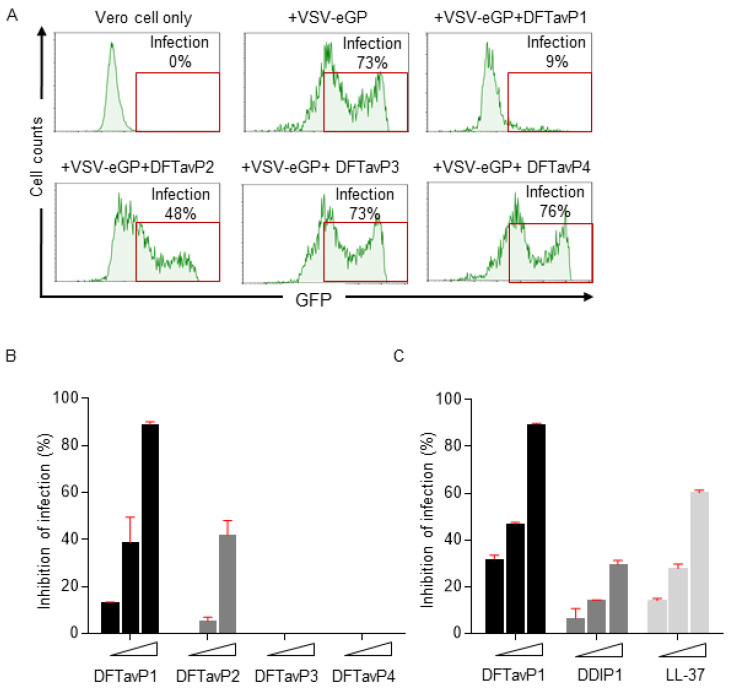
Designed AMPs inhibited the infection of pseudo-EBOV virion in Vero cells. (**A**) AMPs inhibited pseudo-EBOV (VSV-eGP) infection in Vero cells. Vero cells were pretreated with individual AMPs at 10 μM for 2 h before VSV-eGP viruses were added. After 24 h of culture, Vero cells were harvested for flow cytometry analysis to measure viral infection. Percentages of GFP-positive cells represent percentages of cells infected with VSV-eGP. (**B**) DFTavP1 and its derivatives inhibited pseudo-EBOV (VSV-eGP) infection in Vero cells in a dose-dependent manner. Since AMPs usually inhibit microbes at micromolar, Vero cells were pretreated with individual AMPs (at 2.5, 5, or 10 μM) for 2 h before VSV-eGP viruses were added. After 24 h of culture, Vero cells were harvested for flow cytometry analysis to measure viral infection. (**C**) DFTavP1, DDIP1, and LL37 inhibited pseudo-EBOV (VSV-eGP) infection in Vero cells in a dose-dependent manner. Vero cells were pretreated with individual AMPs (at 2.5, 5, or 10 μM) for 2 h before VSV-eGP viruses were added. After 24 h of culture, Vero cells were harvested for flow cytometry analysis to measure viral infection.

The APD, originally established in 2003, was expanded and described in 2009 and 2016 [25,27]. To learn the wisdom of nature, the APD currently focuses on natural peptides with determined antimicrobial activity, known amino acid sequences, and less than 100 amino acids, leading to a widely used core data set. Our careful annotation of peptide activity data (e.g., antibacterial, antiviral, antifungal, antiparasitic, spermicidal, and anticancer) laid a solid foundation for designing peptides with the desired activity. In addition, peptide properties, such as the length, net charge, hydrophobicity, and structure, can be searched for in a systematic manner. Each parameter can be arrayed to identify the optimum. These database features enabled the development of database technology for peptide design. The database filtering technology (DFT) is one such approach that designs new peptides based on the most probable parameters within a set of peptides with common activity [28]. The DFT is an ab initio approach to designing peptides because, unlike de novo approaches, it makes no prior assumptions. The first proposed version of the DFT designed a peptide with activity against methicillin-resistant *Staphylococcus aureus* (MRSA), but did not inhibit Gram-negative bacteria. To design antiviral peptides, the first database filter selected all 190 antiviral peptides in the database (Summer 2021) as templates. These peptides were analyzed to determine the AMP length with the highest abundance. The search was conducted in 10 amino acid increments. The 21–30 residue range had the highest peptide count (Figure 1). This range would be the most probable length for our peptide design. Considering the cost of making longer peptides, however, we selected a peptide length of 20, which was closest to the lower optimal boundary. We then derived the rest of the peptide parameters based on the antiviral peptides with 11–20 amino acids. To study the impact of the AMP length on antiviral activity, we also designed two shorter peptides with 12 and 16 amino acids. In the original DFT design method, a series of filtering steps were involved in deriving numerous parameters. For example, three separate filters were used to determine the frequency of amino acids, net charge, and the hydrophobicity of the anti-MRSA peptide. Here, we found it possible to derive these three parameters in one step by analyzing the amino acid frequency plot for the 11–20-peptide length group. In this plot (Figure 2A), the 20 standard amino acids were separated into four groups based on their common features: (1) hydrophobic (I, V, L, F, C, M, A, and W), (2) special glycine/proline (G and P), (3) polar and hydrophilic (T, S, Y, Q, and N), and (4) charged (E, D, H, K, and R) [25]. We then selected the most abundant amino acid in each group as a representative. In this manner, 20 amino acids were reduced to four for our peptide design (solid columns in Figure 2A, top panel). In this plot, the four representative amino acids were leucine (L), glycine (G), serine (S), and arginine (R). Interestingly, we were not alone in utilizing this reductionist approach, since nature also uses a small set of amino acids to design peptides such as θ-defensins. While the hydrophobic group contained I, V, F, L, and C, the other three groups consisted of a single amino acid (glycine, threonine, and arginine, respectively) (Figure 2B) [29]. This plot indicated that other amino acids in the special GP, polar, and charged groups were not preferred in the known θ-defensins. Next, in our improved design, the amino acid percentages within each amino acid group were summed (Figure 2A bottom panel) to represent the percentage for each of the four selected amino acids (L, G, S, or R) in the designed peptides. To design a 20-mer peptide, we calculated the numbers of the four amino acids L, G, S, and R for this peptide by multiplying the percentage for each amino acid with the targeted peptide length. Thus, this procedure enabled us to determine the types and contents of the four representative amino acids (including hydrophobic and cationic) in one step, increasing the efficiency of this ab initio method. Next, the most probable structure of the peptide was determined to be α-helical, because this structure had the highest occurrence in the 11–20 residue range (Figure 3). This allowed us to place charged and hydrophobic amino acids in a classic amphipathic pattern, where every two leucines were dispersed with two hydrophilic amino acids. To determine the potential combinations of these amino acids in the sequence, a statistical analysis was performed on the possible sequence motifs (four amino acids) that could be formed. The motifs with the highest abundance in the database were selected to connect the leucine pairs. The amino acid sequence of the first peptide (DFTavP1) designed to inhibit viral replication is given in Table 1. For antiviral assays, we utilized a pseudotyped Ebola virus as described previously [24]. In this study, the designed peptides displayed different degrees of inhibitory effects on pseudo-EBOV VSV-eGP (vesicular stomatitis virus-Ebola glycoprotein) infection. DFTavP1, the 20mer, showed an 18% higher inhibition than LL-37 at 2.5 µM (Figure 4). Also, the inhibition increased as the peptide dosage increased.

### 2.2. Validation of the Most Probable Parameters

Next, we validated the improved methodology by designing additional peptides with parameters deviated from the optima. A 16 mer (DFTavP2) was designed in the same manner as DFTavP1 (sequence in Table 1). This peptide length decrease caused a 33% decrease in the viral inhibition of DFTavP2 at 5 µM compared to DFTavP1. A further deviation from the most probable length led to DFTavP3 (a 12 mer in Table 1), which did not inhibit the VSV–eGP (Figure 4B). Hence, the peptide close to the most probable peptide length range was more potent than the sequence-shortened counterparts. 

To further validate the most probable amino acids, leucine in DFTavP2 was converted to valine, leading to DFTavP4 (sequence in Table 1). The flow cytometry results suggested that DFTavP4 entirely lost the ability to inhibit the Ebola pseudo-virus (Figure 4B). This observation indicated the significance of the most probable hydrophobic leucine in conferring antiviral activity to the peptide. The reason for this might be twofold. First, leucine is more hydrophobic than valine, enabling a better binding to viruses. Second, leucine has a higher potential than valine in forming the helical structure required for target binding [25]. To fully validate this, leucine may be converted to other hydrophobic amino acids (I, F, A, M, C, and W) as well. A previous study revealed the peptide became less soluble when substituted by isoleucine [28]. Thus, we did not make the same change. As alanine is even less hydrophobic than valine, we predicted that alanine substitution would also lead to an inactive peptide. We did not test methionine, since this residue is not favorable for peptide design due to its readiness of being oxidized. While phenylalanine and tryptophan substitutions may be of interest for future studies, we included one, W, here for UV quantification. In a different design below, two leucines were transformed to cysteines. 

Additional proof of the most probable principle in peptide design came from our previous database-guided design. The GLK-19 peptide (a 19-residue peptide containing G, L, and K) designed based on the frequently occurring amino acids from amphibian peptides [25] became active to human immunodeficiency virus type 1 (HIV-1) when all lysines were replaced with arginines. These changes were determined based on the arginine/lysine ratios in antibacterial, antifungal, antiviral, and anticancer peptides in the APD, where only in the antiviral peptides was the arginine/lysine ratio greater than one [30]. Since cysteine is also abundant in the hydrophobic group of the antiviral peptide amino acid signature (e.g., see Figure 2A), we changed two leucines in GLR-19 to two cysteines at positions 4 and 16. These changes led to DDIP1 with a disulfide bond. For this study, we created a new version of DDIP1, where all L-amino acids were converted to D-amino acids to gain stability to proteases. Our previous study suggested the importance of peptide stability for inhibiting Ebola viruses [24]. In the inhibition experiment, DDIP1 displayed 7% inhibition at 2.5 µM and 14% inhibition at 5 µM against the virus. Human LL-37, a known antiviral peptide [16,24,30], showed a higher viral inhibition than DDIP1 (Figure 4C). 

### 2.3. Antiviral Efficacy of Peptides Treated before or after Viral Infection 

**Figure 5:**
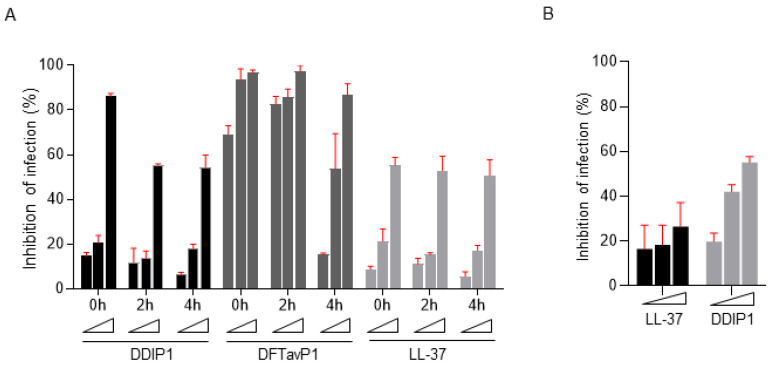
Designed AMPs inhibited EBOV cell entry and SARS-CoV-2 infection. (**A**) AMPs targeted pseudo-EBOV (VSV-eGP) at the early stage of infection. Vero cells were treated with individual AMPs (at 2.5, 5, or 10 μM) at the same time with VSV-eGP (0 h), or at 2 or 4 h after VSV-eGP infection. Vero cells were harvested for flow cytometry analysis of GFP levels at 24 h post infection. (**B**) DDIP1 inhibited SARS-CoV-2 infection. Vero cells were pretreated with individual AMPs (at 2.5, 5, or 10 μM) for 2 h before SARS-CoV-2 viruses were added. After 24 h of culture, Vero cells were harvested for immunofluorescence staining to access infection activity.

We then compared the treatment efficacy of DDIP1 and DFTavP1 postinfection. Human LL-37 was included as a positive control. At 0, 2, and 4 h postinfection, a dose-responsive viral inhibition was observed for all the peptides, indicating the effect results from the peptide treatment (Figure 5A). When treated immediately after infection (delay 0 h), DDIP1, DFTavP1, and LL-37 displayed ~15%, 69%, and 9% inhibitions at 2.5 µM, respectively. Hence, DDIP1 and DFTavP1 demonstrated a higher inhibition than LL-37. DFTavP1, in the 4 h postinfection treatment study, showed a major decrease of 54% inhibition at 5 µM. The inhibition of the Ebola pseudovirus was reduced with a longer delay after infection for both DDIP1 and DFTavP1, implying an action on viral entry. The decrease in inhibition was caused by the decrease in viral load in the extracellular fluid because the intracellular viral load was increasing. Interestingly, the effect of human LL-37 was relatively constant. More experiments are required to determine the precise mechanism of DFTavP1 or DDIP1. 

We also tested the antiviral activity of DDIP1 against SARS-CoV-2. Like LL-37, DDIP1 showed an inhibitory effect on SARS-CoV-2 in a dose-dependent manner (Figure 5B). At 5 µM, LL-37 inhibited 18% of the virus, while DDIP1 suppressed 42%. At 10 µM, DDIP1 (~55% inhibition) was more potent than LL-37 (~27% inhibition) as well. These results reinforced the antiviral potency of the database-designed antiviral peptides. 

### 2.4. Different Requirements for Antiviral and Antibacterial Properties 

As our antiviral peptides were designed based on the antimicrobial peptide database [25], one may wonder whether they are active against bacteria. To obtain a more complete picture, we used two Gram-positive and four Gram-negative bacterial strains and the minimum inhibitory concentrations (MIC) of the peptides are provided in Table 2. In contrast to the antiviral case, DFTavP1 was less active against bacteria than the two shortened peptides DFTavP2 and DFTavP3 (low MIC values). Interestingly, DDIP1 displayed excellent MIC values against all the tested antibiotic-resistant pathogens (e.g., MRSA, *Escherichia coli*, *Pseudomonas aeruginosa*, and *Klebsiella pneumoniae*) in the range of 2–8 µM, comparable to the two shorter DFT peptides (Table 2). These results unveiled different parameter requirements for designing antibacterial and antiviral peptides. We speculate that such a difference primarily resulted from the potential differences in pathogenic targets. It is likely that our designed peptides (e.g., DDIP1) targeted the viral envelope since many AMPs act on bacterial membranes [8,9,10]. Another in silico study screened antiviral peptides by docking known antiviral peptides to the major protease (M^Pro^) of SARS-CoV-2 without experimental validation [31]. Future studies on both viruses and bacteria could validate such mechanisms of action. Our speculation was supported by our previous knowledge on human cathelicidin LL-37 peptides (e.g., GF-17 and GI-20), which inhibit both bacteria and viruses [32]. However, the peptide lost its antiviral effects when the helical structure was disrupted by partially incorporating D-amino acids. In terms of the mechanism, the helical structure of LL-37 peptides was not a must for targeting bacterial membranes, but essential to inhibit HIV-1 reverse transcriptase [33]. In the case of Ebola viruses, only the engineered peptides such as 17BIPHE2 were effective at the endosomal cell entry step by impairing the cathepsin-B-mediated processing of the Ebola viral glycoprotein [34]. 

### 2.5. Cytotoxicity of Antiviral Peptides

For therapeutic use, it is important that the designed peptides had minimal toxic effects on mammalian cells. To evaluate the cytotoxicity of the new peptides, we utilized several cell lines. Vero cells are a model cell for viral infection, derived from the kidney epithelia of the African green monkey. It appeared that Vero cells were highly sensitive to DFTavP1 (TC_50_ 2.4 µM), but became less sensitive to DDIP1 (TC_50_ 13.0 µM in Table 3). Because SARS-CoV-2 infects lung cells, we also tested their cytotoxicity to Calu3 cells. Both DFTavP1 and DDIP1 showed a TC_50_ in the range of 12–14 µM, comparable to the human host defense cathelicidin peptide LL-37 (TC_50_ 19.1 µM). These results indicated a direct antiviral effect of our peptides at a low peptide concentration (e.g., 2.5 µM), where its secondary toxic effect on host cells might play a role. The toxic effect of the designed peptides depended on cell types. The 50% hemolytic concentration (HC_50_) for DDIP1 was greater than 160 µM, the highest concentration we tested (Table 3). However, both DFTavP1 and DFTavP2 were highly hemolytic, with an HC_50_ below 12.5 µM, while DFTavP3, the shortest 12-mer peptide, had an HC_50_ of 50 µM. Likewise, DDIP1 was poorly hemolytic to murine red blood cells as well (HC_50_ > 160 µM), while DFTavP1 was highly hemolytic (Table 3). In the case of skin HaCaT cells, the HC_50_ was 25 µM for DFTavP1, but greater than 100 µM for DDIP1. These results confirmed the toxicity of DFT peptides. However, the engineered peptide DDIP1 was much more selective and showed cell-dependent toxicity (Table 3). 

## 3. Materials and Methods 

### 3.1. The Antimicrobial Peptide Database 

The APD was originally established in 2003. Since then, it has been updated regularly and expanded substantially [25,27]. For scientific rigor, the APD applied a set of criteria for peptide registration (natural peptides, known sequences, known activity, and a size of less than 100 amino acids). Thus, this database currently focuses on natural peptides from six life kingdoms, including bacteria, archaea, protists, fungi, plants, and animals [25]. After over 18 years, 26 types of peptide activities (e.g., antibacterial, antiviral, antifungal, antiparasitic, spermicidal, and anticancer) have been annotated. Such a well-annotated peptide sequence–activity database provides a unique platform for peptide prediction and design. The APD enables a thorough statistical analysis of natural AMPs through a variety of search functions and database filters. Such an analysis identifies key parameters for peptide design [27].

### 3.2. Database Filtering Technology (DFT) vs. Improved DFT

The rigorous registration of the data in the APD set the stage for us to develop database-guided approaches for peptide discovery, ranging from database screening to database filtering technology [27]. The original database filtering technology [28] consisted of multiple database filters that allowed us to derive a family of peptides with desired biological activity, followed by deriving key peptide parameters step by step. In the original DFT, the first filter selected a set of peptides with activity against Gram-positive bacteria, whereas the improved DFT used here selected a group of peptides annotated with antiviral activity. The second filter is common and was used to identify the most probable peptide length. This was achieved by statistically analyzing the peptides in the APD in bins (every 10 s), so that the peptide length with the highest count was found. Subsequently, the original DFT identified the most probable amino acid frequency, net charge, and hydrophobic amino acid in three steps. In contrast, the improved DFT derived frequency, net charge, and hydrophobic content in one step. The rest of the steps in identifying the most probable structure and motifs are shared by both methods and were detailed elsewhere [28]. 

### 3.3. Chemicals and Peptides 

All the chemicals were purchased from established vendors such as Fisher and Sigma. Peptides were created by Genemed Synthesis, Inc. (San Antonio, TX, USA). All the peptides were highly purified and reached over 95% purity based on HPLC. The correct mass of each peptide was validated by mass spectrometry (Shimadzu MALDI-8020, Kyoto, Japan or Thermo Fisher Scientific SALDI-TOF-MS, Waltham, MA, USA). The incorporation of a tryptophan (W) for each peptide at position 2 (in replacement of a leucine in Table 1) facilitated peptide quantification on a UV spectrometer (Ultraspec 1100 pro, Amersham Biosciences) at 280 nm. 

### 3.4. SARS-CoV-2 Safety Statement 

All experiments involving SARS-CoV-2 were conducted in an approved BSL-3 facility of the University of Nebraska Medical Center (UNMC) by dedicated trained personnel. 

### 3.5. Antiviral Assays

Peptide activity against Ebola pseudo-virus was tested using established lab protocols as described [24]. Inhibitory effects on SARS-CoV-2 were tested as below. Live virus experiments were performed in the BSL3 laboratory at the UNMC (Omaha, NE, USA). Briefly, Vero cells (~10,000 cells/well) were seeded in a 96-well plate and cultured in complete medium overnight. In the prevention experiments, cells were pretreated with compounds for 2 h at 37 °C. Treatments were washed off and cells were infected with SARS-CoV-2 WI at a multiplicity of infection (MOI) of 0.1 in complete media. After 24 h, cells were fixed with 4% buffered paraformaldehyde for 30 min at room temperature. The fixed cells were washed with phosphate-buffered saline (PBS), permeabilized with 0.1% (*v*/*v*) Triton X-100 solution for 10 min, then blocked with 3% bovine serum albumin–PBS solution. The cells were incubated with anti-SARS-CoV-2 spike protein rabbit monoclonal antibody (Sino Biological, Beijing, China) at 1:1000 overnight at 4 °C, followed by incubation with 1:2000 diluted Alexa Fluor 488 conjugated secondary antibody (Jackson ImmunoResearch) for 1 h at room temperature. Cell nuclei were counterstained using Hoechst 33,342 (Invitrogen, #H3570), and cytoplasmic membranes were stained with CellMask (Invitrogen, #C10046). Noninfected cells and untreated virus-infected cells were included as internal controls. Cells were imaged using a high-content analysis system, Operetta CLS (PerkinElmer Inc., Waltham, MA, USA). Percentage inhibition of viral infection was calculated using Harmony 4.9 software (PerkinElmer Inc.).

### 3.6. Antibacterial Assays

Peptide activity against bacteria was tested using established lab protocols as described previously [28]. In brief, a peptide concentration gradient with two-fold dilution was created in the 96-well polystyrene microplates at 10 μL per well. From overnight cultures, six bacteria (Table 2) were grown to the logarithmic phase (i.e., optical density at 600 nm ≈ 0.5), diluted to ~10^5^ CFU/mL, and partitioned into the 96-well microplates at 90 μL per well. The microplates were incubated at 37 °C overnight and read on a ChroMate 4300 Microplate Reader at 600 nm (GMI, Ramsey, MN, USA). 

### 3.7. Cytotoxicity 

Peptide toxicity was evaluated by using human red blood cells (hRBCs) and other host cells. Hemolysis was conducted as described elsewhere [28]. Briefly, hRBCs, obtained from the UNMC Blood Bank, were washed three times with phosphate-buffered saline (PBS) and diluted to a 2% solution (*v*/*v*). After peptide treatment, incubation at 37 °C for one hour, and centrifugation at 13,000 rpm, aliquots of the supernatant were carefully transferred to a fresh 96-well microplate. The amount of hemoglobin released was measured at 545 nm. The percent lysis was calculated by assuming 100% release when human blood cells were treated with 2% Triton X-100, and 0% release when incubated with PBS buffer. The peptide concentration that caused 50% lysis of hRBCs was defined as HC_50_. 

Vero cells (ATCC, CCL-81) or Calu-3 (ATCC, HTB-55) cells were seeded into 96-well tissue culture plates (Greiner Bio-One, Monroe, NC) and treated with different concentrations of peptides for 24/48 h at 37 °C. Cell viability was examined by Vybrant^®^ MTT Cell Proliferation Assay Kit (Thermo Fisher Scientific, Grand Island, NY) following the manufacturer’s instructions. Toxicity assays using HaCaT cells were conducted similarly as described elsewhere [34]. 

## 4. Conclusions

It is useful to identify antiviral peptides that can eliminate all kinds of SARS-CoV-2 variants: alpha, beta, delta, omicron, etc. Based on the antimicrobial peptide database [25], we previously developed a database filtering technology [28], which was recently proved to be useful for us in the design of anti-MRSA peptides with systemic efficacy in mice [34]. Here, we improved the original stepwise database filtering method by deriving multiple most probable parameters in a single amino acid composition analysis for designing antiviral peptides, thereby accelerating this peptide design process based on the antimicrobial peptide database. Our designed peptides were indeed inhibitory to the Ebola pseudo-virus. In addition, both human cathelicidin LL-37 and DDIP1 could inhibit SARS-CoV-2. The decrease in or loss of activity of peptides created with deviated most probable parameters further validated the improved method. Our study demonstrated that the designed peptides were inhibitory to the Ebola pseudo-virus in either prevention or treatment experiments. The reduced antiviral effect of the designed peptides after additional delay postinfection implied the blockage of viral entry. Our engineered disulfide-containing peptide DDIP1 with numerous desired properties could be further optimized to remove toxicity and developed into a novel treatment for viral infections such as COVID-19. This should require the study of pharmacokinetics and pharmacodynamics of the optimized peptide in animal models. It is predicted that DDIP1 is effective against a variety of mutated viral strains, since it may damage viral envelopes to prevent infection in a similar manner to disrupt bacterial membranes. Our discovery of novel antiviral peptides may be further accelerated by applying the machine learning/artificial intelligence algorithms [35,36,37,38,39,40,41,42,43] that enable the prediction of both the antiviral activity and toxicity of peptides with the accumulation of experimental data of AMPs. 

## Figures and Tables

**Table 1:** Database-designed antiviral peptides and their properties.

Peptide	Amino Acid Sequence ^a^	Length	Net Charge	Pho ^b^	Boman Index ^c^	GRAVY
DFTavP1	RWLRGLLSGLLRRLLSGLLL	20	+5	55	0.6	0.815
DFTavP2	RWLRGLLSGLLRRLLS	16	+5	50	1.73	0.331
DFTavP3	RWLRGLLSGLLR	12	+4	50	1.61	0.25
DFTavP4	RWVRGVVSGVVRRVVS	16	+5	50	2.12	0.506
DDIP1 ^d^	GLRCRLGRLLRRLGRCLLR	19	+7	47	3.4	−0.0579

^a^ All the peptides were C-terminally amidated except for DDIP1. A tryptophan was introduced into the DFT peptides to facilitate peptide quantification (see Methods section). ^b^ Hydrophobic content. ^c^ Boman index (originally called protein binding potential in kcal/mol) [8] was renamed by the APD in 2003 [25]. ^d^ All were D-amino acids. In addition, a disulfide bond exists between the two cysteines C4 and C16 of DDIP1.

**Table 2:** Minimum inhibitory concentration (µM) of database-designed antiviral peptides.

Peptide	*S. aureus* USA300	*Staphylococcus epidermidis* 1457	*Escherichia coli* E416-7	*Pseudomonas aeruginosa #2*	*Klebsiella pneumoniae* E406-17	*Acinetobacter baumannii* B28-16
DFTavP1	8–16	8–16	16	32	16–32	8
DFTavP2	4	2	4	8	4	2
DFTavP3	4	8	2–4	4–8	8	4
DFTavP4	>64	NA	16	NA	NA	NA
DDIP1	4	4–8	2	4	4	2–4

**Table 3:** Cytotoxicity comparison of antiviral peptides to different cells.

Peptide	hRBC HC_50_ ^a^	mRBC HC_50_ ^b^	HaCaT TC_50_ ^c^	Vero Cell TC_50_	Calu3 Cell TC_50_
DFTavP1	<12.5 µM	<10 µM	25 µM	2.39	11.88
DDIP1	>160 µM	>160 µM	>100 µM	13	14.25

^a^ hRBC, human red blood cells; ^b^ mRBC, BALB/c mouse red blood cells; ^c^ TC_50_, the peptide concentration that killed 50% of human keratinocytes.

## Data Availability

Publicly available datasets were analyzed in this study. This data can be found here: https://aps.unmc.edu (accessed on 1 June 2021) [44].
